# Evaluation of CD47 in the Suppressive Tumor Microenvironment and Immunotherapy in Prostate Cancer

**DOI:** 10.1155/2023/2473075

**Published:** 2023-09-09

**Authors:** Qianqian Wang, Chunxaing Feng, Yuchun Chen, Tianming Peng, Yong Li, Kunlin Wu, Xiaoyong Pu, Hanzhong Chen, Jiumin Liu

**Affiliations:** ^1^Department of Urology, School of Medicine, South China University of Technology, Guangzhou 510006, China; ^2^Department of Urology, Guangdong Provincial People's Hospital (Guangdong Academy of Medical Sciences), Southern Medical University, Guangzhou 510080, China

## Abstract

**Background:**

CD47 has high levels of expression in malignant cancer cells, which binds to SIRP-*α* to release the “don't eat me” signal and prevents mononuclear macrophages from phagocytosing the cells. Resistance to drugs and metastases are potential barriers for prostate cancer endocrine therapy. Although immunotherapy for tumors has developed rapidly in the last few decades, its effectiveness in treating prostate cancer is unsatisfactory. Prostate cancer has a high-expression level of CD47. Therefore, a novel approach for potential immunotherapy may be provided by investigating the relationship among CD47 and the infiltration of immune cells in the prostate carcinoma.

**Methods:**

The GEPIA database was utilized to compare the abundance of CD47 in malignant tissues with tissues that were normal. Furthermore, the function of CD47 in prostate carcinoma was assessed by CancerSEA. The association among CD47 and the tumor microenvironment was assessed utilizing the TISCH single cell data database. By using TIMER, the connection among CD47 and immunological invasion of prostate cancer was explored. Moreover, macrophages were cocultured with mouse prostate cancer cell RM-1 blocked by CD47 antibody to observe the changes in phagocytosis efficiency *in vitro*.

**Results:**

Expression level of CD47 is upregulated in prostate carcinoma, and it is closely connected with prostate cancer's inadequate immune invasion. CD47 antibody blocking promotes macrophage phagocytosis of RM-1.

**Conclusion:**

Our research demonstrates a closely relationship among CD47 and the immunological microenvironment of prostate cancer, and blocking CD47 can promote macrophages to phagocytosis of prostate cancer cells. Therefore, CD47 may provide novel strategies for potential immunotherapy of prostate cancer.

## 1. Introduction

Prostate cancer (PCa), the second most prevalent tumor and the fifth most common cause of cancer mortality in men worldwide, will also be the primary reason for newly diagnosed cases of cancer and the second-most typical common reason for cancer-related death in males in the United States by 2023 [1, 2]. In China, the incidence of prostate cancer is increasing year by year, with 30% of newly diagnosed patients showing distant metastases [3]. The main treatment for advanced prostate cancer is androgen deprived therapy (ADT). Metastatic castration-resistant prostate cancer (mCRPC) eventually develops in patients using ADT as well [4]. Nowadays, immunotherapy is an important part of treatment strategies for patients suffering from advanced malignancies. Because it has the capacity to stimulate the immune system of the tumors, which ultimately helps suppress tumor growth and prevent metastasis and recurrence [5]. With few mutations and several immune escape mechanisms, PCa is referred to as a “cold tumor” in immunology [6]. The effectiveness of immune checkpoint inhibitors (ICIs) has been examined in numerous clinical investigations. However, ICIs showed limited antitumor activity in early studies [7]. In metastatic prostate cancer patients, myeloid suppressor cells and regulatory T cells showed increased suppressive phenotypes in circulating and tumor microenvironments (TME), and immune suppressive TME were enhanced [8]. Immune suppressive TME pose challenges for effective immunotherapy.

Different from PD-1/PD-L1 induced acquired immune response mediated by cytotoxicity T lymphocytes, as a part of innate immunity, macrophages phagocytose foreign substances without the need to express specific signaling proteins on the cell surface. Tumor-associated macrophages (TAMs) can accelerate or slow the progression of cancer by regulating the inflammatory response in TME [9]. Absence or abnormality of innate immune response is a crucial cause of tumor immune evasion and treatment resistance. Activation of innate immunity can significantly increase the effectiveness and response rate of immunotherapy. Studies have shown that the use of four different immune stimulation methods to activate the innate and acquired immune systems of tumor-forming mice at the same time can promote the body's immune system to produce strong synergistic killing effect of tumor cells, and even achieve complete digestion of highly invasive advanced tumor tissues [10]. However, the immune system can be circumvented by tumor cells in a variety of ways, one of which is by upregulating integrin-associated protein (IAP-) CD47 [11]. Transmembrane protein CD47 is a member of the immunoglobulin superfamily [12]. It is widely expressed on the cell surface, but overexpressed on many different tumor cells. It attaches to signal-regulatory protein-*α* (SIRP-*α*) on phagocytes to evade phagocytic effect on tumor cells [13]. According to studies, suppressing the CD47 signal activates the immune system and encourages macrophages to phagocytose mouse melanoma cells [14]. However, there is minimal information available about CD47's role in prostate cancer TME. Therefore, this study used GEPIA to examine the variation in CD47 expression among carcinoma and normal tissues, TISCH and TIMER databases to explore the connection among CD47 and prostate cancer immunoinfiltrating cells, and *in vitro* phagocytosis experiments were conducted to block CD47 signals. The objective of this study is to look into the connection among CD47 and immune cell infiltration in prostate tumor, with the goal of providing a fresh strategy for treating prostate cancer.

## 2. Materials and Methods

### 2.1. Analysis of Differential Expression

GEPIA (http://gepia.cancer-pku.cn/), a web service for gene expression analysis and interaction analysis in cancer and normal samples, includes RNA sequencing expression information gathered from 9,736 malignancies and 8,587 normal tissue samples obtained from the TCGA and GTEx databases [15]. The difference among the expression levels of CD47 in normal and neoplastic tissues was examined using GEPIA. CD47 was entered into the search box on the homepage of the website to analyze its expression in various malignancies.

### 2.2. Analysis of Correlation

CancerSEA (http://biocc.hrbmu.edu.cn/CancerSEA/), a dedicated database for the analysis of different functional states of various tumor cells at the single-cell level [16]. Input CD47 in the search box and limit the functional relevance in prostate cancer. Filter by correlation strength > 0.1, and *p* value < 0.05. Online research was done to examine the relationship among CD47 and prostate cancer using the CancerSEA database.

### 2.3. Immunoinfiltration Analysis

TISCH (http://tisch.comp-genomics.org/) united the data sets by including 79 databases and 2,045,746 cells from carcinoma patients as well as donors who were healthy [17]. The relationship between CD47 and the percentage of cell invasion in the TME of the original and metastatic prostate cancer was examined using the TISCH dataset. In the Dataset field of the TISCH website, the tumor type was selected as prostate adenocarcinoma, and the data sets containing metastases were screened for analysis.

TIMER (http://timer.comp-genomics.org/timer/) utilized RNA-Seq expression profile data to find the infiltration of immune cells in tissues from tumors [18]. It was utilized to examine how CD47 and the quantity of immunoinfiltrating cells in prostate cancer relate to one another.

### 2.4. Materials and Cell Line

aCD47, PE anti-mouse CD11b, PE/Cyanine7 anti-mouse CD80, and APC anti-mouse F4/80 were bought from Biolegend (cat. no. 127517, cat. no.101208, cat. no.104734, and cat. no.123116). M-CSF was purchased from Sino Biological (cat. no. 51112-MNHA). Lipopolysaccharides (LPS) were purchased from Sigma (cat. no. L4391). RM-1, purchased from Procell, is an epithelial-like adherent cell from murine prostate cancer. In an incubator set to 37°C with 5% CO_2_, the DMEM (Gibco) supplemented with 10% fetal bovine serum (Excell Bio) and 1% penicillin–streptomycin (Gibco) was utilized for cultivating the cells.

Male Balb/c mice (6–10 weeks) were bought from Guangdong Medical Laboratory Animal Center. The Guangdong Provincial People's Hospital's Ethics Committee has approved this trial.

### 2.5. Immunofluorescence Staining

A total five pairs of tumor tissue specimens and normal specimens were collected from patients diagnosed with prostate adenocarcinoma in Guangdong Provincial People's Hospital. All tissues were sectioned after paraffin embedding, and HE staining was used to identify the tumor region. In accordance to the manufacturer's directions, tumor sections were cut, placed on the slides, and stained by using two different primary antibodies, including CD47 (Santacruze, cat. no. SC-12730) and CD163 (Abcam, cat. no. ab182422), overnight at a temperature of 4°C. Subsequently, fluorescently labeled secondary antibodies—goat anti-rabbit IgG (H + L; Abcam, cat. no. ab150088) and goat anti-mouse IgG (H + L; Abcam, cat. no. ab150117)—were added to the slides. CD47 was diluted 50 times, and other antibodies were diluted 200 times. Fluorescence signal was observed under the fluorescence microscope (Nikon).

### 2.6. Assay for Phagocytosis

Bone marrow derived macrophages (BMDM) were extracted and cultivated according to the protocol of Ying et al. [19]. Bilateral tibia and femur of mice were taken, both ends of the bone were cut. The cells were separated from the bone marrow using a 10 mL syringe and serum-free media. To get rid of cell clumping, run the cell through a 70 *μ*m cell strainer. Red blood cells (RBC) can be eliminated by including 4 mL of red blood cell lysis buffer (Solarbio, cat. no. R1010) and incubating for 5 min on ice. 500× g for 5 min is used for spinning down cells. Finally, the cells were suspended at 1×10^6^ cells/mL after being given two PBS washes. Additionally, complete medium containing 20 ng/mL M-CSF was used to inoculate the cells in the cell plate. After 7 days of induced differentiation, mouse BMDM was taken, and trypsin was added for digestion and cell collection. After centrifugation, the cells were twice washed with PBS prior to being suspended 1×10^6^ cells per tube, followed by the addition of CD11b (Biolegend, cat. no. 101208), CD80 (Biolegend, cat. no. 104734), and F4/80 (Biolegend, cat. no. 123116) and a 20 min incubation period on ice away from light. All of the antibodies utilized for the tests were 200 times diluted. Utilizing CytoFLEX flow cytometry (Beckman), the stained cells were examined while the nonstaining group served as a blank control.

BMDM were activated by treating with 100 ng/mL LPS for 24 hr. Activated BMDM were stained with PKH67, and RM-1 were labeled with Dil. Macrophages (1 × 10 ^5^) were cocultured with RM-1 cells that had been previously inhibited with IgG or aCD47 for 2 hr at 37°C. Phagocytosis was observed *in vitro* under the inverted fluorescence microscope and CytoFLEX flow cytometry 2 hr later.

### 2.7. Statistical Analysis

The significance of unpaired *t* test was analyzed using GraphPad Prism 9 software (GraphPad), and the measurement results were reported as the mean SD, with a *p* value < 0.05 denoting a statistically significant difference.

## 3. Results

### 3.1. CD47 Expression Level in Prostate Cancer

**Figure 1:**
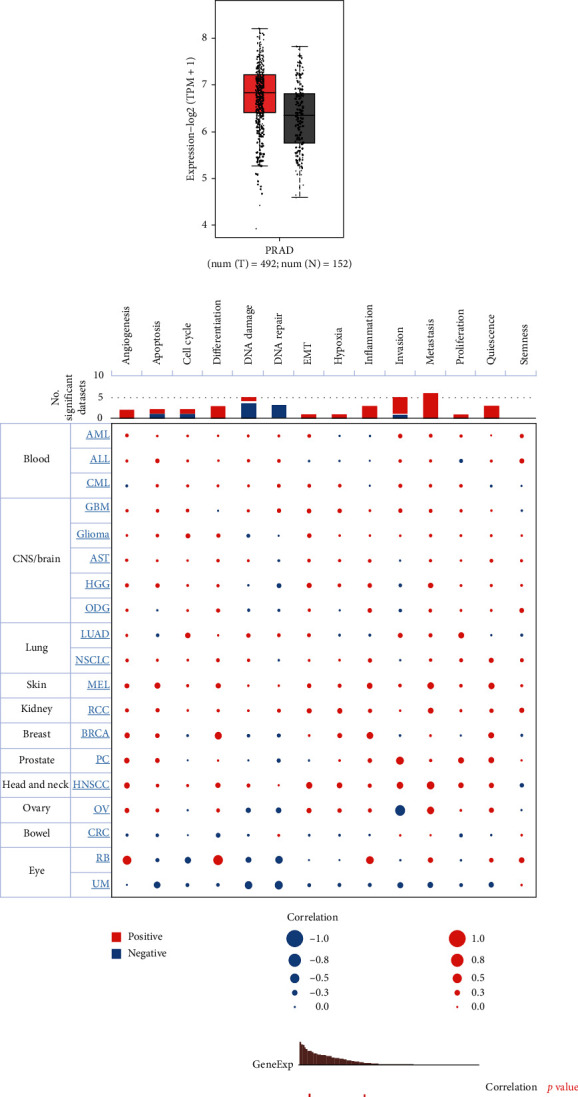
Expression and functional characterization of CD47 in prostate cancer by GEPIA and CancerSEA databases. (a, b) CD47 expression is elevated in prostate cancer tissues. (c–e) Functional characterization of CD47 in prostate cancer.

The level of expression of CD47 in both benign and cancerous prostate tissues was investigated using GEPIA. The level of expression of CD47 was elevated in prostate tumors, as illustrated in Figures 1(a) and 1(b). The relationship among CD47 and prostate cancer was further examined using the CancerSEA database. The correlation between CD47 and different functional states of various cancers is shown in Figure 1(c). CD47 was associated with prostate cancer invasion, angiogenesis, resting, and proliferation (Figures 1(d) and 1(e)). These results suggest that CD47 is highly expressed in prostate carcinoma and plays a part in a number of pathological and physiological processes.

### 3.2. Relationship between the Tumor Microenvironment and CD47

**Figure 2:**
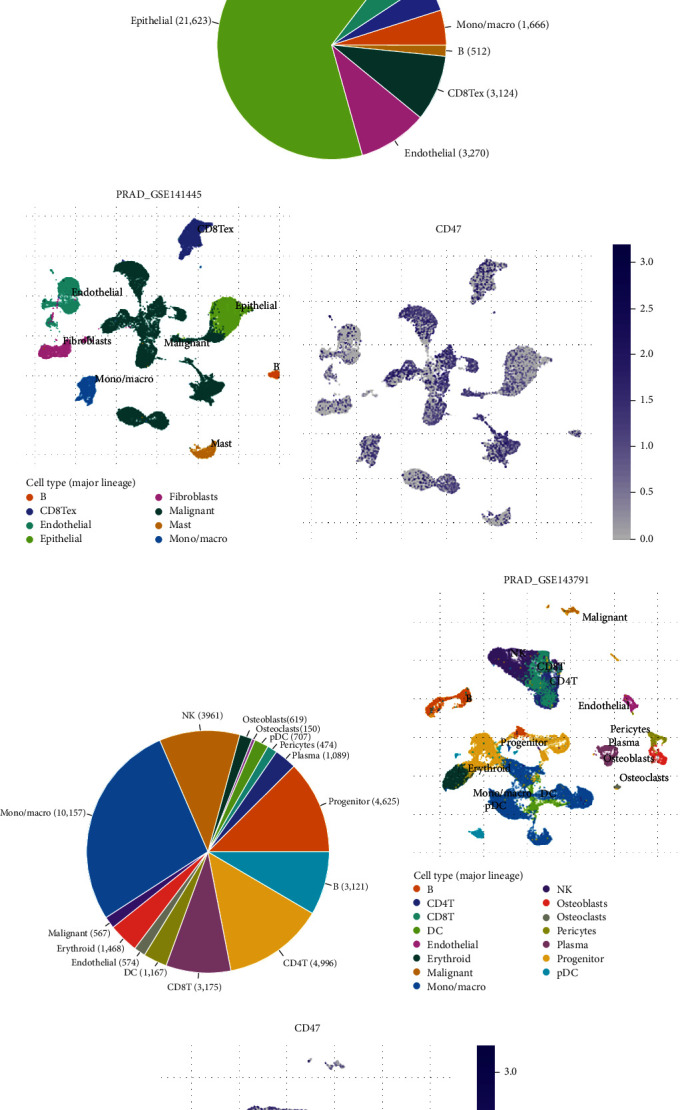
Relevance between CD47 and tumor microenvironment. (a) CD47 was expressed differently in different cells from different data sets. (b–d) Levels of CD47 expression in diverse immune cell populations in the PRAD-GSE141445 dataset. (e–g) Levels of CD47 expression in diverse immune cell populations in the PRAD-GSE143791 dataset.

Using the TISCH database, the expression of CD47 in immune cells related to TME was assessed at the single-cell level. The levels of expression of CD47 were low in various immune-related cells such as mast cells and endothelial cells in multiple data sets, while high in malignant cells (Figure 2(a)). This indicates that CD47 expression level is high in malignant prostate cancer cells. Two data sets containing distant metastasis, PRAD-GSE141445 and PRAD-GSE143791, were then screened for comparison. The PRAD-GSE141445 dataset included 12 patients with primary prostate cancer and 1 patient with lymph node metastasis [20]. The PRAD-GSE143791 dataset included bone marrow single cell data from 9 patients with prostate cancer bone metastasis and 7 patients with hip replacement [21]. The result shows PRAD-GSE141445 was mainly in epithelial cell, and that malignant cells and CD8+T cells expressed CD47 at a higher level than other cell types (Figure 2(b)–2(d)). PRAD-GSE143791 showed a richer variety of cells, as well as different expression levels of CD47 in different immune cells (Figure 2(e)–2(g)). These findings suggest that CD47 expression levels vary between various cell types and are higher in malignant cells.

### 3.3. Prostate Cancer Immune Invasion Is Correlated with CD47 Expression

**Figure 3:**
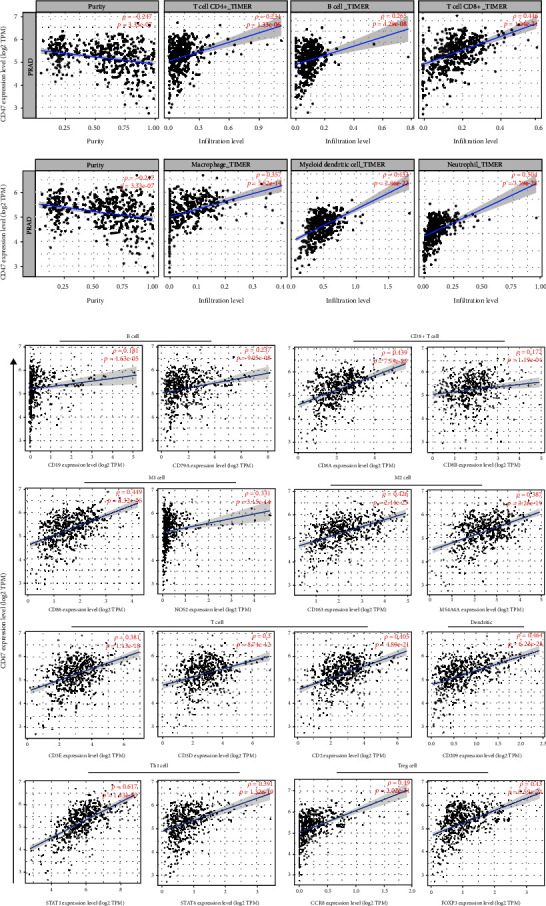
Analysis of correlation between CD47 and infiltration of immune-related cells in prostate carcinoma. (a) The relevance of CD47 and immune cells infiltration. (b) Analysis of the association among the CD47 and immune cell marker genes.

**Figure 4:**
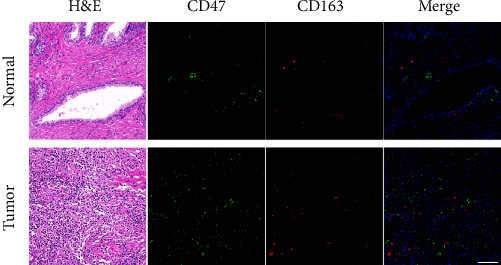
Spatially distribution and accumulation of CD47 and macrophages in prostate cancer tissues. Representative H&E image and immunofluorescence images of CD163 (red) and CD47 (green) in normal and tumor prostate tissue. Scale bar, 100 *μ*m.

The immune infiltration degree of prostate cancer was explored utilizing the TIMER database for further investigate the connection among CD47 and the immunological microenvironment of prostate cancer. Positive correlations between CD47 expression and B cells, CD8+T cells, macrophages were found, while negative correlation among CD47 expression and tumor purity were found (Figure 3(a)). In addition, immunocell markers were used to evaluate CD47 expression, and the outcomes demonstrated that CD47 was related to T cells, M2 cells, and Treg cells, while the correlation with B cells was lower than previously reported (Figure 3(b)). In all the samples we collected, high expression of CD47 was connected to CD163+ macrophage infiltration (Figure 4).In summary, the expression of CD47 may regulate TME through immune cell infiltration, thus regulating tumor progression.

### 3.4. CD47 Antibody Blocking Promotes Macrophage Phagocytosis of Tumor Cells

**Figure 5:**
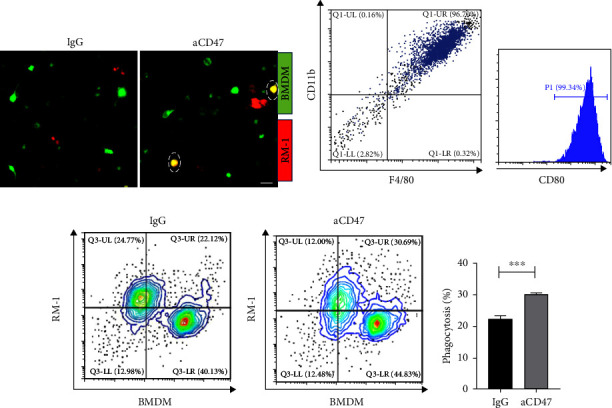
*In vitro* phagocytosis test. (a) Fluorescent representations of the RM-1 cells (in red) that have been phagocytosed and the macrophage (in green). Scale bar, 50 *μ*m (b, c) Original BMDM identification. (d, e) Phagocytosis ratio of experimental group and control group was analyzed by flow cytometry, *p* < 0.01. Statistical analyses were performed applying unpaired *t* test.

To further verify the impact of CD47 as a potential target on prostate cancer TME, we used aCD47 antibody to block RM-1 and observe the phagocytosis efficiency of M1-like macrophages on it. The bone marrow cavity of the mouse tibia was used to obtain primary BMDM. As shown in Figure 5(a), the proportion of mø cells induced by M-Csf was 96.7%, which proved that mø BMDM was successfully induced (Figure 5(b)). Then, LPS was used to induce M1-type macrophages, and the proportion of CD80+ cells was 99.34% (Figure 5(c)). This indicated that M1 macrophages were successfully induced.

Then, RM-1 was blocked with aCD47 and cocultured with M1 BMDM to observe the phagocytosis ratio. By using a fluorescent microscope, the cellular phagocytosis was investigated. The IgG group's macrophages showed minimal red RM-1 fluorescence, whereas the aCD47 group's fluorescence was significantly higher. Furthermore, flow cytometry data demonstrated that the phagocytosis ratio of the IgG blocked group was 22.34% and that of the aCD47 blocked group was 30.2%, which was 31% greater than the rate of the control group (Figures 5(d) and 5(e)), and *p* < 0.01. In conclusion, aCD47 can promote phagocytosis of M1-like macrophages by blocking RM-1.

## 4. Discussion

The TME of PCa is intricate, which exhibit highly interconnected and complex signaling networks, creating a favorable ecological environment for the survival, development, and metastasis of tumors [22]. As a “cold” tumor, PCa responds poorly to the regular immune therapies currently used [23]. In the majority of malignancies, high-macrophage infiltration is linked to a negative outcome [24]. Under physiological conditions, CD47 on normal RBC binds to SIRP-*α* of macrophages to produce signals of inhibition that prohibit phagocytosis. When RBC age, CD47 expression is reduced, and the aged red blood cells are treated as foreign cells by the immune system and are rapidly removed by the macrophages in the spleen [25]. In contrast to noncancerous bone specimens, Mohanty et al. [26] discovered that human osteosarcoma samples had greater levels of CD47 expression. Besides, in gastric carcinoma, abnormal CD47 expression is a standalone indicator of adverse survival outcomes and fluorouracil-based adjuvant chemotherapy resistance [27]. We discovered that CD47 has high levels in prostate carcinoma and that it is positively connected with prostate cancer invasion, angiogenesis, resting, and proliferation and negatively correlated with DNA repair based on our examination of data from the GEPIA and CancerSEA databases. Multiple investigations have proven that CD47 has an impact in a wide range of physiological activities, including immune system homeostasis, nitric oxide signaling suppression, cell migration, apoptosis, and phagocytosis [28]. Within peripheral tissues, the presence of CD47 on T cells plays a vital role in promoting T cell viability and maintaining the efficacy of the adaptive immune system. The absence of CD47 significantly diminishes the T cell population in peripheral tissues, and T cells lacking CD47 exhibit necrosis upon interaction with conventional dendritic cells. Therefore, CD47 could serve as a viable immunological candidate for prostate cancer [29].

To learn more about how immune cell expression and CD47 relate to the microenvironment of prostate cancer, TISCH database was used for single-cell level analysis. The findings demonstrated that CD47 expression levels differed between multiple kinds of immune-related cells and were higher in the cancerous cells. The PRAD-GSE141445 dataset containing metastatic prostate cancer showed high expression of CD47 in CD8+T cells while low expression in B cells. The findings demonstrated that CD47 expression levels differed among various kinds of immune cells and were higher in the cancerous cells. According to these findings, immune cell infiltration in prostate carcinoma may be linked to CD47. Additionally, we discovered that CD47 had a strong association with the infiltration of CD8+T cells but less association with the involvement of B cells. Spatial heterogeneity is brought on by different immune cell recruitment and localization patterns in the prostate cancer microenvironment from lesion to lesion [30]. CD4+T cell infiltration take part in the development and spread of prostate cancer [31], and neutrophils are linked to poor prognosis [32].Therefore, we proposed that the expression level of CD47 might affect TME through immune cell infiltration, thus regulating tumor progression.

The level of immune-related cell penetration and tumor type are two variables that may have an impact on how an immunotherapy affects a tumor's response [33]. The prognosis of a tumor may be correlated with tumor infiltration of immune cells [34]. According to Burugu's research, immune cell density and dispersion may have an impact on the outcome of breast cancer [28]. TAMs account for a large part of tumor immune infiltrating cells [35]. It can be differentiated into antitumorigenic M1-like phenotype and protumorigenic M2-like phenotype [36]. M1-like TAM is involved in tumor antagonism, and studies have revealed that M1-like TAM may be positively correlated with patient survival [37]. M2-like TAM invasion is usually associated with tumor invasion, migration, angiogenesis, T-cell inhibition, and adverse clinical outcome [36]. M2-like TAMs and Treg cells can produce an immune barrier response [38]. In prostate tumor tissue, a prior investigation revealed that the amount of M2-like TAMs increased considerably [21, 39]. Using the TIMER database to investigate the infiltration of immune-related cells within prostate carcinoma, it was discovered that M2-like TAMs and Treg cell invasion proportions also increased in the prostate cancer microenvironment with high-CD47 expression. And immunofluorescence staining of our tissue samples suggested that positive expression of CD163, which is often utilized as an identifier for M2-like TAMs, was associated with high expression of CD47 [40]. Patients with PCa had a nearly fivefold higher risk of recurrence if they had significant levels of M2-macrophage infiltration [41]. In a number of malignancies, M2-like TAMs have been found to suppress immune responses against tumors [42]. Based on the sonodynamic therapy and the characteristics of macrophage apoptosis induced by zoledronic acid, Cao et al. [43] constructed M2-like TAMs targeted nanoliposomes by using M2-pep, a polypeptide sequence that tends to bind M2-like TAMs, which effectively consumed M2-like TAMs, alleviated tumor hypoxia, increased the release of immune-promoting factors, and achieved antitumor effects. Increasing evidence shows that TME can change the properties of macrophages to maintain dynamic tissue homeostasis, and targeting tam has become a promising immunotherapy strategy in the field of solid tumors [44, 45]. Given the plasticity of macrophages, macrophages at different locations within the same tumor may receive different signals from their immediate microenvironment and develop different functions [46]. Different approaches can therefore be taken to harness them for therapeutic purposes, such as elimination or inhibition of tumor-promoting macrophages, expansion or activation of antitumor macrophages, and conversion of subtypes or any combination thereof [47].

Blocking CD47 decreases tumor burden *in vivo* and *in vitro*, as demonstrated by numerous models and clinical investigations [48–50]. In our studies, *in vitro* phagocytosis test confirmed that the inhibition of RM-1 by aCD47 antibody can promote the phagocytosis of M1-like phenotype macrophages. Studies have also demonstrated that blocking CD47 can reduce tumor load. Targeted CD47-Sirp-*α* signaling also has multiple mechanisms of action in antitumor therapy. By activating CD8+T cells and dendritic cells, anti-CD47 antibodies prompt these cells to engulf cancerous cells and process their unique antigens. They then deliver those antigens to CD8+T cells, which in turn stimulate a tumor-specific adaptive immune response that effectively kills tumors [11]. Apart from enhancing macrophage phagocytosis of tumor cells, blocking CD47 may also increase macrophage recruitment to tumor cells. However, blocking CD47 can transform TAMs into antitumor state and enable more macrophages to recruit to the tumor [51, 52]. Therefore, prostate cancer may be treated with tumor treatments that target CD47. These findings suggest that varying kinds of immune cells express CD47 at various levels, and an elevated level of expression is linked to immunological invasion of prostate cancer. Therefore, the elevated level of CD47 may promote the immune escape of prostate cancer cells, thus affecting the prognosis.

## 5. Conclusion

In summary, our findings suggest that the level of immune infiltration of prostate cancer is connected with the expression level of CD47, and blocking CD47 *in vitro* can promote macrophages to phagocytosis of prostate cancer cells. Therefore, CD47 might be crucial in the immunoinfiltration of prostate cancer microenvironment, which will provide ideas for the potential function of CD47 in the immunotherapy of prostate cancer.

## Data Availability

All experimental data used to support the findings of this study are available from the corresponding author upon request.
